# Internal Urethrotomy Under Local Urethral Anaesthesia Is Feasible With Sedation and Analgesia

**DOI:** 10.5812/numonthly.4524

**Published:** 2012-09-24

**Authors:** Hakki Uzun, Orhan Ünal Zorba, Yakup Tomak, Habip Bostan, Mehmet Kalkan

**Affiliations:** 1 Department of Urology, School of Medicine Rize University, Rize, Turkey; 2 Department of Anaesthesiology, School of Medicine Rize University, Rize, Turkey; 3 Department of Urology, Özel Sema Hastanesi, Istanbul, Turkey

**Keywords:** Urethral Stricture, Internal Urethrotomy, Anaesthesia

## Abstract

**Background:**

Urethral stricture is a common condition, and direct vision internal urethrotomy is prefered as the first treatment option by many urologists, for strictures shorter than 2 cm. This procedure is generally performed under general or spinal anaesthesia.

**Objectives:**

To investigate the feasibility of adding local urethral anaesthesia to intravenous sedation and analgesia (sedoanalgesia) methods in patients undergoing internal urethrotomy.

**Patients and Methods:**

A total of 21 and 15 patients with anterior urethral strictures underwent internal urethrotomy under local urethral anaesthesia, with or without sedoanalgesia, respectively. Patient discomfort and pain levels were evaluated using the visual analog scale (VAS). Statistical analyses were calculated with a Mann-Whitney U test to compare difference in VAS scores between the subjects in both groups.

**Results:**

Two of the 15 (13%) patients operated under local urethral anaesthesia without sedoanalgesia were converted to general anaesthesia due to patient intolerability. Mean pain VAS scores for patients operated under 2% lidocain urethral gel anaesthesia with or without sedoanalgesia were 2.86 cm and 4.5 cm, respectively (P = 0.001). In addition, a VAS score over 3 cm was found in 3 of the 21 (14%) patients with, and 13 of the 15 (86%) patients without sedoanalgesia (P = 0.001).

**Conclusions:**

The addition of intravenous sedoanalgesia improved the VAS scores of pain and discomfort, compared to patients operated under only local urethral anaesthesia. This may offer patients safer anaesthesia and shorter operative times with equilavent results in selected patients.

## 1. Background

Urethral stricture is a common condition and direct vision internal urethrotomy is prefered as the first treatment option by many urologists for strictures shorter than 2 cm, this procedure is generally performed under general or spinal anaesthesia (1). Nevertheless, various local anaesthesia techniques, including topical anaesthesia (2-6), spongiosum block (7), transperineal (8), and urethrosphincteric blocks (9), have been used, but none of these methods have gained wide acceptance. Due to the short operative time and the frequency of the procedure among endourological interventions, a less invasive and feasible anaesthesia technique will continue to be in demand.

## 2. Objectives

We investigated the feasibility of the addition of intravenous sedoanalgesia to local urethral anaesthesia in patients undergoing an internal urethrotomy.

## 3. Patients and Methods

Between December 2009 and January 2011, an internal urethrotomy was performed in 36 patients with anterior urethral strictures shorter than 2 cm. Patients were prospectively seperated into two groups, including 21 and 15 patients who were operated under local urethral lidocain gel anaesthesia with and without intravenous sedoanalgesia, respectively. The average age of the 15 patients in the local urethral anaesthesia group was 69.41 years and the average age of the 21 patients operated under local urethral anaesthesia with the addition of intravenous sedoanalgesia was 63.73 years The urologists selected the patients nonrandomly for the anaesthesia condition. The two different options for anaesthesia were offered to all patients and the anaesthesia method was applied according to the patient's choice.

Anatomic urethral location, estimated stricture length under urethroscopic vision, potential cause of stricture, and previous urethral interventions were all recorded. All anterior urethral strictures were solitary lesions, primary, and uncomplicated. Patients with multiple and posterior strictures were excluded. The etiology of the urethral stricture was determined as; traumatic, inflammatory or unknown. The diagnosis of urethral stricture was based on; clinical symptoms, medical history, physical examination, blood and urine tests, ultrasonography of the urinary tract and uroflowmetry.

A flexible urethroscopy was performed to confirm the diagnosis preoperatively in all patients. Since flexible ureteroscopy is our standard diagnostic procedure for suspected urethral strictures, we do not routinely perform a retrograd urethrogram. We also did not need to perform a urodynamic study on the patients. The length of the strictures were calculated to be between 1 and 1.5 cm in each of the groups due to estimations made during the cystoscopy. The operations were postponed until the urine became sterile. Exclusion criteria were; uncorrected coagulopathy, allergy to opioids, history of drug or alcohol abuse, posterior urethral stricture, multiple strictures or strictures longer than 2 cm.

Internal urethrotomy was performed in the operating room. Patients who received intravenous sedaoanalgesia were monitorized continuously throughout the procedure with; electrocardiography, blood pressure, heart rate, and peripheral oxygen saturation (SpO_2_) measures. Supplemental oxygen 3 L/min was administered with a facemask. The patient was set up for the operation in the lithotomy position. In the first group of patients, 10 mL 2% lidocaine gel was instilled into the urethra and retained by a penile clamp for 10 minutes. The other group also received 2% lidocain gel anaesthesia, but in addition, 0.03 mg/kg midazolam and 1 μg/kg fentanil were administered intravenously 10 minutes after the instillation of lidocain gel and before the start of the urethrotomy. Operative time was calculated from the insertion of the urethrotome to the urethral foley catheterization. Multiple incisions were performed at 12, 3 and 9 o’clock positions with a 20 Fr cold-cutting urethrotome (Olympus, Japan) under direct vision. A 14 to 16 F foley urethral catheter was inserted for 4 to 5 days. Antibiotics were taken only on the day of the operation. All patients were treated on an outpatient basis. Patients were discharged on the same day and followed up for at least one year.

At the end of each operation in the operating room, patients were asked to scale their level of discomfort and/or pain experienced during the procedure using a 10-point linear visual analog pain scale (VAS). A VAS score between 1 and 3 was considered acceptable and regarded as mild pain, and scores over 3 were considered to be unacceptable as an alternative to spinal or general anaesthesia which are approved for moderate pain. A written consent form was taken from each patient and the local Ethics Commitee of our university approved the study.

### 3.1. Statistical Analysis

A Mann-Whitney U test was used to compare differences in VAS scores between subjects operated under local urethral anaesthesia with or without sedoanalgesia. All statistical tests were two-tailed. A P value < 0.05 was considered statistically significant. All analyses were performed using PASW version 18.0 (SPSS Inc., Chicago, IL, USA).

## 4. Results

The mean operative time for the group without sedoanalgesia was 18.5 minutes and 12.6 minutes for the group with intravenous sedoanalgesia. Internal urethrotomy was successfully performed in all patients in both groups, but two patients who were operated under local urethral anaesthesia without sedoanalgesia were converted to general anaesthesia due to patient intolerability. At follow up, three and four patients in the local urethral anaesthesia and sedoanalgesia groups, respectively, had a recurrence of stricture and were considered to be treatment failures. Repeat urethrotomies and subsequent urethral dilations were performed. No anaesthesia-related complications or urinary infections developed.

The patients operated under local urethral anaesthesia with sedoanalgesia found the pain more tolerable. Mean pain VAS scores for patients operated under 2% lidocain urethral gel anaesthesia with or without sedoanalgesia was 2.86 cm and 4.5 cm respectively. In comparison to lidocain gel anaesthesia, the addition of intravenous sedoanalgesia significantly decreased the discomfort (P = 0.001). Additionally, a VAS score over 3 cm was found in 3 of 21 (14%) and 13 of 15 (86%) of patients in favour of local urethral anaesthesia with sedoanalgesia (P = 0.001) (Table 1). No patients, other than the patients converted to general anaesthesia, reported a score of over 7 in either group. The etiologic classification of urethral strictures is shown in Table 2.

**Table 1:** Average Age, Mean Operative Time and VAS Scores in Both Groups

	Local Urethral Anaesthesia	Local Urethral Anaesthesia + Sedoanalgesia
**No. of Patients**	15	21
**Age (mean)**	69.41	63.73
**Mean procedure time (min)**	18.5	12.46
**VAS Score**	4.5	2.86
**Mild pain (VAS 1-3), (%)**	2 (14)	18 (86)
**Moderate pain (VAS 4-7) , (%)**	13 (86)	3 (14)
**Conversion to general anaesthesia, (%)**	2/15 (13)	0

**Table 2:** Etiology of Urethral Strictures

	Local Urethral Anaesthesia, No. (%)	Local Urethral Anaesthesia + Sedoanalgesia, No. (%)
**Traumatic**	4 (27)	5 (24)
**Inflammatory**	6 (40)	8 (38)
**Unknown**	5 (33)	8 (38)

## 5. Discussion

Internal urethrotomy is frequently preferred in short urethral strictures, as it can be done as an outpatient procedure, with short operative times. Lidocain gel local urethral anaesthesia has been studied, but conflicting results have been revealed (2-6). Most of these trials have reported that the procedure was well tolerated, but the anaesthesia method has not found acceptance in clinical practice. Krede et al., however, reported that three of the 18 patients under topical anaesthesia failed due to severe pain (2). Additionally, Ye et al. reported that under local urethral anaesthesia, urethrotomy resulted in severe, sharp pains during the incision of the fibrous scar tissue and the majority of the patients were not able to tolerate the discomfort (7). Our experience also showed, that the peak moment of the operation for pain under local urethral gel anaesthesia, is the cutting of the fibrous scar tissue. In addition, many patients need multiple incisions to advance the urethrotome into the bladder and that might increase the severity of the pain and discomfort. In our study 86% of the patients in the local urethral anaesthesia group had moderate pain (VAS score > 3 cm), that is to say, we believe that it is inadvisable for patients to change from the alternative of spinal anaesthesia. On the other hand this rate was only 14% in the sedoanalgesia group, therefore, we think that local urethral anaesthesia with the addition of sedoanalgesia is feasible for selected patients. Furthermore, we believe as the length of the stricture decreases, the pain or discomfort might also be lower.

Ye et al. proposed intracopus spongiosum anaesthesia for urethral interventions and reported satisfactory results with low pain scores (7). However, injection into the glans penis may cause severe pain compared to intramuscular injection. Additionally, they advised a slow injection of lidocaine into the glans to avoid instantaneous trance. Unfortunately, the slow injection process prolongs the procedure time and this might lead to an increase in pain and discomfort. Furthermore, thinking of the injection entering into the glans might cause anxiety in the preoperative period and this could result in many patients preferring other anaesthesia alternatives.

Local anaesthesia with sedoanalgesia for urethrotomy has several advantages compared to spinal or general anaesthesia. The anxiety associated with general anaesthesia is eliminated with sedation. Midazolam has been reported to decrease anxiety preoperatively and postoperatively without any significant effect on vital signs (10). In addition, the risks of postoperative nausea and headache are eliminated with our protocol. Moreover, patients who have high risk factors could be safely managed with a local urethral anaesthesia combined with sedoanalgesia. Sedoanalgesia also has a shorter anaesthesia preperation and recovery time, which might translate into lower costs. Intravenous alfentanyl and midazolam has been shown to be safe and efficient for many endourological procedures with a 46% decrease in costs (11). Aside from the cost benefits, the patients can return to their daily activities earlier.

There may be concern about patient discomfort in the case of extention in the duration of the operation. However, the incidence of long operative times is low and there is always the potential to turn the operation into a spinal or general anaesthesia. Nevertheless, our protocol is not advisable for operations with long urethral strictures and/or those requiring a long time period of surgery.

The addition of intravenous sedoanalgesia to local urethral anaesthesia improved VAS pain scores and provided the surgeons with a greater feeling of confidence against patient discomfort during the procedure. Our protocol might also offer patients safer anaesthesia and shorter operative times with equilavent results. Furthermore, an internal urethrotomy could be performed in an office setting, in selected cases.
